# Effectiveness of a healthy lifestyle intervention for low back pain and osteoarthritis of the knee: protocol and statistical analysis plan for two randomised controlled trials

**DOI:** 10.1590/bjpt-rbf.2014.0189

**Published:** 2016-09-22

**Authors:** Kate M. O’Brien, Amanda Williams, John Wiggers, Luke Wolfenden, Serene Yoong, Elizabeth Campbell, Steven J. Kamper, James McAuley, John Attia, Chris Oldmeadow, Christopher M. Williams

**Affiliations:** 1 Hunter New England Population Health, Wallsend, NSW, Australia; 2 Hunter Medical Research Institute, Hunter Region Mc, NSW, Australia; 3 School of Medicine and Public Health, University of Newcastle (UON), Newcastle, Australia; 4 The George Institute for Global Health, University of Sydney, Sydney, NSW, Australia; 5 Neuroscience Research Australia, Randwick, NSW, Australia

**Keywords:** low back pain, knee osteoarthritis, lifestyle, telephone, randomised controlled trial, statistical analysis plan

## Abstract

**Background:**

These trials are the first randomised controlled trials of telephone-based weight management and healthy lifestyle interventions for low back pain and knee osteoarthritis. This article describes the protocol and statistical analysis plan.

**Method:**

These trials are parallel randomised controlled trials that investigate and compare the effect of a telephone-based weight management and healthy lifestyle intervention for improving pain intensity in overweight or obese patients with low back pain or knee osteoarthritis. The analysis plan was finalised prior to initiation of analyses. All data collected as part of the trial were reviewed, without stratification by group, and classified by baseline characteristics, process of care and trial outcomes. Trial outcomes were classified as primary and secondary outcomes. Appropriate descriptive statistics and statistical testing of between-group differences, where relevant, have been planned and described.

**Conclusions:**

A protocol for standard analyses was developed for the results of two randomised controlled trials. This protocol describes the data, and the pre-determined statistical tests of relevant outcome measures. The plan demonstrates transparent and verifiable use of the data collected. This *a priori* protocol will be followed to ensure rigorous standards of data analysis are strictly adhered to.

## BULLET POINTS

- Lifestyle factors such as overweight and obesity are associated with low back pain and osteoarthritis. However, accessible interventions aiming to support patients with low back pain or osteoarthritis to manage lifestyle factors have not been tested in high quality trials.
- The two trials determine the effectiveness of telephone-based healthy lifestyle interventions for low back pain and osteoarthritis of the knee.
- This protocol comprehensively describes key trial methodology relating to data capture, management and pre-determined statistical analyses.
- Such protocols are important in raising the validity of physical therapy research as they demonstrate transparent and verifiable use of the data collected and ensure rigorous standards of data analysis are strictly adhered to.

## Introduction

This protocol describes the first randomised controlled trials (RCT) of telephone-based weight management and healthy lifestyle interventions for low back pain and knee osteoarthritis. Here we describe the protocol and pre-determined statistical analysis plan, for both trials (trial one: *low back pain* and trial two: *knee osteoarthritis*). The protocol and statistical analysis plan was finalised prior to analysing the data and will be adhered to in analysing the data from the trials. All study investigators signed and approved the statistical analysis plan in May 2016. Participant recruitment for both trials was completed in October 2015, and final participant follow-up was completed in May 2016. Following data integrity checks the database will be locked (June 2016). The statistical analyses specified in the statistical analysis plan will be performed in June 2016.

## Study overview

### Study design and setting

These trials were established as part of a cohort multiple RCT design^1^, whereby participants from our existing cohort of patients referred for an outpatient orthopaedic consultation at a public tertiary referral hospital within NSW Australia, were randomised to be offered a new clinical intervention (intervention group) or remain as part of the cohort (control group). Both trials were prospectively registered with the Australian New Zealand Clinical Trials Registry (trial one: ACTRN12615000478516, and trial two: ACTRN12615000490572) and full study protocols for each trial have been published elsewhere^2^ ^,^ ^3^. These trials were approved by the Hunter New England Health Human Research Ethics Committee (13/12/11/5.18), Wallsend NSW, Australia and the University of Newcastle Human Research Ethics Committee (H-2015-0043), Newcastle, Australia.

### Participants and recruitment

Patients with non-specific low back pain (trial one, n=160) or knee osteoarthritis (trial two, n=120) were recruited. Participants in the intervention group of both trials were provided with brief advice and education about the benefits of weight loss and physical activity for their conditions by trained telephone interviewers. Additionally, participants in the intervention group of trial one (low back pain) were provided with an initial consultation with the study physical therapist. The consultation involved a low back pain clinical assessment and detailed low back pain education based on clinical practice guidelines. Behavioural change techniques were also utilised to support a healthy lifestyle and weight management for low back pain. There was no baseline clinical assessment for participants of trial two (knee osteoarthritis).

Following baseline data collection, participants in the intervention groups of both trials were referred to the NSW Get Healthy Information and Coaching Service (GHS). The GHS is a free, telephone-based government funded service to support individuals to modify their eating behaviours, increase their physical activity, reduce alcohol consumption and achieve or maintain a healthy weight^4^. The GHS has been shown to be effective in the general population and involves 10 individually tailored coaching calls delivered over a 6-month period by a university-qualified health professional. The support provided is based on national guidelines and utilises motivational interviewing^4^. All health coaches were provided with training by a study investigator (CW) in evidence-based management for low back pain and knee osteoarthritis.

Participants in the control group received any usual care offered to them by their treating clinician during the six month intervention period, and participated in data collection. Follow-up lasted for 26 weeks (6 months).

### Inclusion/exclusion criteria

Patients were eligible for inclusion in the trials if all of the following criteria were met:

• Trial one condition definition: chronic low back pain defined as pain in the lower back (i.e. between the 12th rib and buttock crease) with/without leg pain and duration of longer than 3 months since the onset of pain^5^;
• Trial two condition definition: complaint of pain in the knee due to knee osteoarthritis (as per referral) lasting longer than 3 months;
• Aged 18 years or older;
• Classified as overweight or obese with a self-reported body mass index (BMI) ≥27kg/m^2^ and <40kg/m^2^;
• Have access to and can use a telephone; and
• Have back or knee pain, for each trial respectively, severe enough to cause at least average pain intensity ≥3 of 10 on a 0–10 numerical rating scale (NRS)^6^ in the last week or moderate level of interference in activities of daily living (adaptation of item 8 on SF36)^7^.

Patients were excluded if they met the following criteria:

• Known or suspected serious pathology as the underlying cause of back pain or knee osteoarthritis, for each trial respectively, (e.g. fracture, cancer, infection, inflammatory arthritis, infection, cauda equine syndrome);
• A previous history of obesity surgery;
• Current participation in any prescribed, medically supervised or commercial weight loss program;
• Back or knee surgery, for each trial respectively, in the last 6 months or booked in for surgery in the next 6 months;
• Unable to walk unaided;
• Unable to comply with the study protocol that requires them to, adapt meals or exercise, due to non-independent living arrangements;
• Any medical or physical impairment, apart from back pain or knee osteoarthritis for each trial respectively, precluding safe participation in exercise such as uncontrolled hypertension, or morbid obesity (BMI≥40); and
• Unable to speak and read English sufficiently to complete the study procedures.

### Unblinding

The analysis plan was written and approved prior to analysis of data and blind to group status. Dummy coded variables representing group allocation will be used to ensure blinding of statistician(s) undertaking the analysis.

## Objectives

The primary objective of both trials is to establish if:

Trial one: pain education and referral to a telephone-based weight management and healthy lifestyle intervention improves pain intensity in patients with *low back pain*, who are overweight or obese, compared to usual care.

Trial two: referral to a telephone-based weight management and healthy lifestyle intervention improves pain intensity in patients with *knee osteoarthritis*, who are overweight or obese compared to usual care.

Secondary aims of the two trials is to establish if the telephone interventions lead to reductions in disability, weight, BMI, waist circumference, alcohol consumption, and smoking prevalence, and improvement in quality of life, emotional distress, sleep quality, physical activity, diet, pain attitudes and beliefs, perceived change in condition and change in health care and medication use.

A separate analysis plan will be detailed for health economic analyses and is not included in this manuscript.

### Definition of outcome variables

#### Participant demographics and baseline characteristics

Baseline data includes: age, gender, Aboriginal and/or Torres Strait Islander status, employment status, country of origin, highest level of education, health insurance status, other co-existing medical conditions needing medication, and pain duration (how long have you been troubled with your pain). Length of time waiting for consultation (days) and triage classification will be obtained from hospital records. In Australia, patients referred for orthopaedic consultation are categorised according to urgency of consultation: urgent – to be seen within 30 days; semi-urgent – to be seen within 90 days; and non-urgent – to be seen within 12 months^8^. See Table 1 for details.

**Table 1:** Demographic and baseline characteristics.

	**Intervention**	**Control**
**Demographic**		
Age (years)	mean (SD)	mean (SD)
Gender (male)	n/N (%)	n/N (%)
Aboriginal and/or Torres Strait Islander status	n/N (%)	n/N (%)
Employment status		
Employed	n/N (%)	n/N (%)
Unemployed	n/N (%)	n/N (%)
Retired	n/N (%)	n/N (%)
Can’t work (health reasons)	n/N (%)	n/N (%)
Country of origin (Australia)	n/N (%)	n/N (%)
Highest level of education		
>High school	n/N (%)	n/N (%)
Private health insurance	n/N (%)	n/N (%)
Other co-existing medical conditions needing medication	n/N (%)	n/N (%)
Length of time waiting for consultation (days)	mean (SD)	mean (SD)
Triage classification		
Non-urgent	n/N (%)	n/N (%)
Semi-urgent	n/N (%)	n/N (%)
**Baseline characteristics**		
Pain intensity (NRS)	mean (SD)	mean (SD)
Pain duration (how long have you been troubled with your pain)	mean (SD)	mean (SD)
Disability and function (Trial 1: RMDQ / Trial 2: WOMAC)	mean (SD)	mean (SD)
Subjective weight	mean (SD)	mean (SD)
BMI	mean (SD)	mean (SD)
Quality of Life (SF12.v2)		
Physical component score (PCS)	mean (SD)	mean (SD)
Mental component score (MCS)	mean (SD)	mean (SD)
Emotional distress (DASS-21)	mean (SD)	mean (SD)
Poor sleep quality (item 6, Pittsburgh Sleep Quality Index)	n/N (%)	n/N (%)
Physical activity (mins MVPA/week)	mean (SD)	mean (SD)
Diet		
Fruit (serves)	n/N (%)	n/N (%)
Vegetables (serves)	n/N (%)	n/N (%)
Discretionary foods (serves)	n/N (%)	n/N (%)
Alcohol consumption (AUDIT)	mean (SD)	mean (SD)
Smoking prevalence	n/N (%)	n/N (%)
Pain attitudes (SOPA)	mean (SD)	mean (SD)
Fear avoidance beliefs (FABQ)	mean (SD)	mean (SD)
Health care utilisation		
Medication use for back or knee pain	n/N (%)	n/N (%)
Visits for back or knee pain	n/N (%)	n/N (%)

NRS=numerical rating scale; RMDQ=Roland Morris Disability Questionnaire; WOMAC=Western Ontario and McMaster Universities Index; BMI=Body Mass Index; SF12.v2= Short Form Health Survey version 2; PCS=Physical Component Score; MCS=Mental Component Score; 21=Depression Anxiety Stress Scale; MVPA=Moderate-to-Vigorous Physical Activity; AUDIT=Alcohol Use Disorders Identification Test; SOPA=Survey of Pain Attitudes; FABQ=Fear Avoidance Beliefs Questionnaire.

#### Primary outcome

The primary outcomes are average weekly back pain intensity (trial one) and average weekly knee pain intensity (trial two), measured over the course of follow up.

Participants were asked to report the “average pain intensity experienced in their back (trial one) or knee (trial two) over the past week, on a 0 to 10 NRS, where 0 was ‘no pain’ and 10 was the ‘worst possible pain’”^6^. These pain intensity scores were measured at baseline, at 2, 6, 10, 14, 18, 22 and 26 weeks. Average weekly (back or knee) pain intensity is defined as the Area under the Curve (AUC) of the pain intensity trajectory, over the follow up period. The AUC for each participant will be computed using the trapezoid rule.

#### Secondary outcomes

The secondary outcomes include:

• Physical disability and function, measured in trial one using the Roland Morris Disability Questionnaire (RMDQ)^9^ 0-24 scale and measured in trial two using the Western Ontario and McMaster Universities Osteoarthritis Index (WOMAC) 0-96 scale^10^;
• Self-reported weight (kg);
• Objective weight (kg) measured to the nearest 0.1kg by a trained research assistant using International Society for the Advancement of Kinanthropometry (ISAK) procedures^11^;
• BMI calculated as weight /height squared (kg/m^2^)^12^;
• Waist circumference measured by a trained research assistant using ISAK procedures taken at the level of the narrowest point between the inferior rib border and the iliac crest using a flexible tape measure to the nearest 0.1 cm^11^;
• Quality of life, measured using the physical and mental health component scores from the 12-item Short Form Health Survey version 2 (SF12.v2)^7^;
• Global perceived change in symptoms, measured using the Global Perceived Effect (GPE) scale (-5 ‘vastly worse’ to 5 ‘completely recovered’)^13^;
• Emotional distress, measured using the Depression Anxiety Stress Scale-21 (DASS-21) 0-63 scale^14^;
• Sleep quality, measured using item 6 from the Pittsburgh Sleep Quality Index (response options: very bad, fairly bad, fairly good, very good)^15^;
• Physical activity, measured using the Active Australia Survey^16^, reported as the average minutes spent participating in moderate-to-vigorous physical activity (MVPA) per week;
• Diet, measured using a short food frequency questionnaire (FFQ)^17^, reported as serves of fruit (0-1, 2 or more), serves of vegetables (0-2, 3-4, 5 or more), serves of discretionary foods including processed meats, salty snacks, takeaway meals, sweet or savoury snacks, confectionary and sugar sweetened beverages (more than once per week, once per week or less);
• Alcohol consumption measured using the Alcohol Use Disorders Identification Test (AUDIT) 0-12 scale^18^;
• Smoking prevalence (have you smoked any tobacco in the last 4 weeks? (this can include cigarettes, roll your own, pipes, cigars or any other tobacco products))^19^;
• Attitudes and beliefs, measured using the Survey of Pain Attitudes (SOPA)^20^; and the physical component of the Fear Avoidance Beliefs Questionnaire (FABQ) 0-24 scale^21^; and
• Health care utilisation for each trial respectively, including back or knee pain medication use (name), type of health service utilised for back or knee pain including number of sessions, and attended orthopaedic consultation or received surgery.

See Table 2 for data collection time points for secondary outcomes.

**Table 2:** Secondary outcome measures.

**Construct**	**Measurement**	**Time-point (weeks)**
Disability and function	Trial one: Roland Morris Disability Questionnaire (RMDQ)^9^ Trial two: Western Ontario and McMaster Universities Osteoarthritis Index (WOMAC)^10^	0, 6, 26 0, 6, 26
Subjective weight	Self-reported weight (kg)	0, 6, 26
Objective weight	Measured to the nearest 0.1kg^11^	0^a^, 26
BMI	BMI calculated as weight/height squared (kg/m^2^)^12^	0, 6, 26
Waist circumference	Measured to the nearest 0.1cm^11^	26
Quality of life	Short Form Health Survey version 2 (SF12.v2)^7^	0, 6, 26
Perceived change in condition	Global Perceived Effect scale (–5 to 5 scale)^13^	6, 26
Emotional distress	Depression Anxiety Stress Scale-21 (DASS-21)^14^	0, 26
Sleep quality	Item 6 from the Pittsburgh Sleep Quality Index^15^	0, 6, 26
Physical activity	The Active Australia Survey^16^	0, 6, 26
Diet	Short food frequency questionnaire^17^	0, 6, 26
Alcohol consumption	Alcohol Use Disorders Identification Test (AUDIT)^18^	0, 6, 26
Smoking prevalence	Self-reported current smoking status^19^	0, 6, 26
Pain Attitudes	Survey of Pain Attitudes (SOPA)^20^	0, 6, 26
Fear avoidance beliefs	Fear Avoidance Beliefs Questionnaire (FABQ)^21^	0, 26
Health care utilisation	Medication use for back (trial one) or knee pain (trial two) Visits for back (trial one) or knee pain (trial two) – type and number of sessions Attended orthopaedic consultation, received surgery	0, 6, 26 0, 6, 26 26

a Intervention group of low back pain patients (trial one) only. BMI: Body Mass Index.

### Process variables

#### Intervention fidelity

Delivery of the intervention is assessed by the GHS, data includes; commencement, the number, length, and timing of coaching calls and achievement of identified goals.

#### Concomitant treatments

Participants were asked to record separately all medication and health care services used for the back or knee pain, for each trial respectively, at baseline, and weeks 6 and 26 post-randomisation. Information for each additional treatment was provided as free text often using variable terminology. These will be aggregated using a common terminology. Medications will be coded using the Anatomical Therapeutic Chemical Classification System at the third level. Other health services will be coded according to common provider types, for example specialist, hospital or emergency department presentation or admission, physical therapy, chiropractic, massage therapy, other allied health, alternative medicine, and other.

#### Safety

Participants were monitored for adverse events throughout the intervention period. All adverse events (AE), that is, any new medical conditions or an exacerbation of another existing condition, were recorded at 6 and 26 weeks. All AEs will be described for each group.

### Design issues

#### General design

These trials were parallel group RCTs, established as part of a cohort multiple RCT. Patients waiting for an outpatient orthopaedic consultation at a public tertiary referral hospital within NSW were sent an information letter to invite participation in the cohort (telephone survey) and again at 12-months follow-up. At 12-month follow-up patients consenting to the telephone survey were screened for eligibility for the RCT by a trained interviewer and invited to participate if eligible for the study.

#### Treatment allocation

Eligible patients were randomised in a 1:1 allocation ratio, to either receive the weight management and healthy lifestyle intervention at that time (intervention group) or remain as part of the cohort and be told they will be offered clinical services in 6 months (control group). The randomisation schedule was generated a priori by an independent statistician using SAS 9.3 through the SURVEYSELECT procedure. To randomise patients, a trained interviewer opened a sealed opaque envelope containing group allocation. A staff member not involved in the study prepared the envelopes.

#### Sample size

The sample size for both trials was calculated using Stata sample size calculator.

For trial one a standard deviation of 2.3, a two-sided alpha of 0.025 (to account for two outcomes of interest, the primary outcome (pain) and the key secondary outcome (weight)^22^ and allowing for 15% loss to follow-up was used. A sample size of 80 participants per group (n=160) has 90% power to detect a clinically meaningful difference of 1.5 points in pain intensity (pain NRS) between intervention and control groups^23^. This sample also provides power 80% to detect a 6% reduction in weight in the underlying sampling population, based on evidence from other musculoskeletal conditions this is hypothesised to lead to a clinically meaningful reduction in pain^23^.

For trial two a standard deviation of 2.7, a two-sided alpha of 0.025 (to account for two outcomes of interest, the primary outcome (pain) and the key secondary outcome (weight)^22^ and allowing for 15% loss to follow up, a sample of 60 participants per group will provide 90% power to detect a clinically meaningful difference of 2 points in pain intensity (pain NRS) scores between intervention and control groups at 26 weeks. This sample also provides 80% power to detect a 6% weight reduction which is hypothesised to be lead to a clinically meaningful reduction in pain^23^.

In these calculations the increase in statistical power conferred by reducing error variance through repeated outcome measures over time and the correlations among repeated measures have been conservatively ignored.

#### Data collection and follow up

The different stages of data collection and follow-up for secondary outcomes are summarised in table one. The primary outcome, pain intensity score, was collected at baseline, week 2, 6, 10, 14, 18, 22 and at 26 weeks. Baseline assessment was conducted prior to randomisation.

#### Interim analysis

No interim analysis was conducted.

## Statistical analysis

### Trial profile

Flow of the patients through the study will be displayed in a Consolidated Standards of Reporting Trials (CONSORT) diagram for each trial. We will report the number of screened patients who met study inclusion criteria, reasons for exclusion of non-included patients, the number of participants randomised per group, and the number who completed follow-up, as shown in Figures 1A and 1B.

**Figure 1A:**
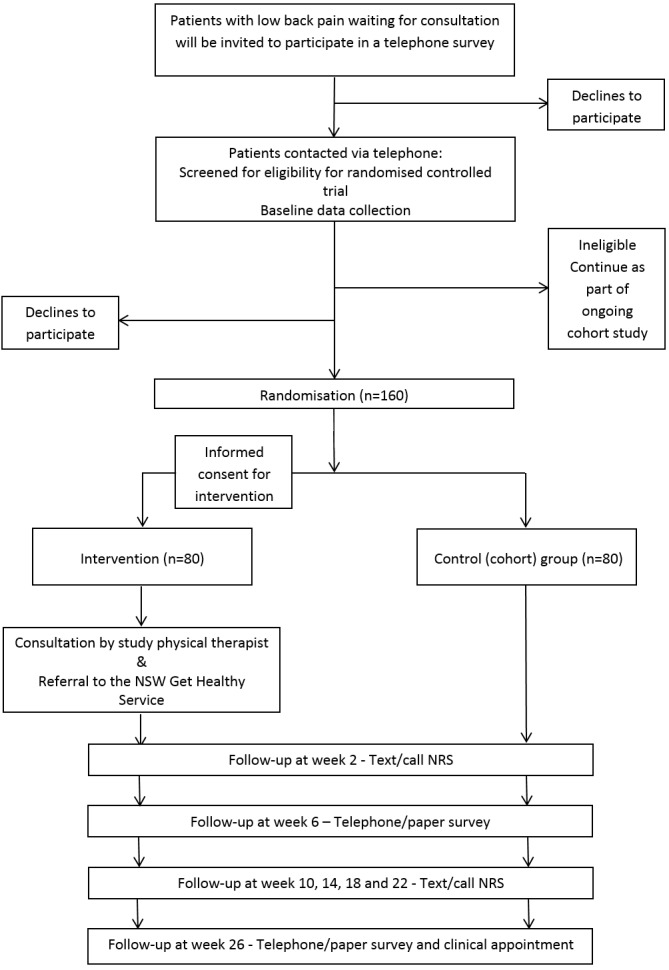
Progress of participants through trial one (low back pain).

**Figure 1B:**
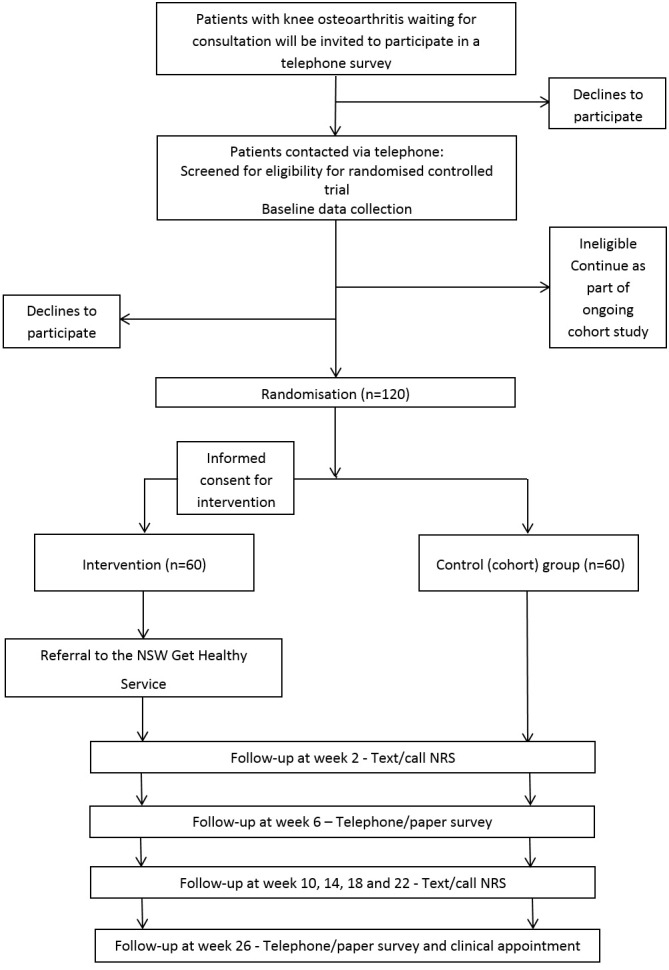
Progress of participants through trial two (knee osteoarthritis).

### Data integrity

Trial data integrity will be monitored by regularly scrutinising data files for data omissions and errors. Manually entered data (i.e. data not recorded directly by the participant) will be double entered and the source of any inconsistencies will be explored and resolved in consultation with the lead investigator (CW).

### Analysis principles

Primary analyses will be conducted independently by an independent statistician who is blinded to group status.

Analyses will be conducted using SAS V9.4 (SAS Institute, Cary, North Carolina, USA). Intention-to-treat (ITT) (analysed as randomised) will be utilised. All statistical tests will be two-tailed. Treatment effect for the primary and secondary outcomes will be considered significant if *p*≤0.025 and *p*≤0.01, respectively.

Summaries of continuous variables that are symmetrically distributed will be presented as means and standard deviations (SD) or medians and inter-quartiles for skewed data, whereas categorical variables will be presented as frequencies and percentages. Large count variables will be reported as medians and interquartile ranges, low counts (max count <5) will be presented as frequencies and percentages.

### Analysis population

The ITT population is defined as all randomised participants with a baseline measurement. Participants failing to record an outcome value at any follow-up period will be treated using the methods described below (see “Methods for handling missing data”).

### Methods for handling missing data

The number of participants with missing observations will be reported for each outcome variable. Patterns of missing data will be investigated and compared by demographic characteristics of the participants, t-tests will be used to compare continuous variables and chi-square tests will be used to compare categorical variables. For the primary outcome variable (average weekly pain intensity score) for participants with <10% missing pain intensity values, the missing pain intensity values will be interpolated using cubic spline interpolation. For participants with 10% or greater missing data an AUC will not be computed. The primary method of dealing with missing AUC data will be through multiple imputation (assuming missing at random), where missing AUC data will be imputed using the chained equations method of generating a number of complete data sets; the imputation model will include a range of covariates believed to be associated with either the missing outcome or the outcome itself (baseline pain and duration, waiting time, BMI). Sensitivity of analysis results will be assessed by comparing results obtained various imputation models. If there is reason to suggest the data may be missing not at random, pattern mixture models will be utilised^24^.

### Evaluation of demographics and baseline characteristics

The description of baseline characteristics listed below will be presented by treatment group. Categorical variables will be summarized by frequencies or denominators and percentages. Percentages will be calculated using the number of patients for whom data is available as the denominator. Denominators will be systematically reported (for example, nn/NN, %). Continuous variables will be summarised using standard measures of central tendency and dispersion, either mean and SD, or median and interquartile range.

- Age at randomisation
- Gender
- Aboriginal and/or Torres Strait Islander
- Employment status
- Country of origin
- Highest level of education
- Private health insurance
- Other co-existing medical conditions needing medication
- Length of time waiting for consultation (days)
- Triage classification
- Pain intensity and duration
- Disability and function
- Subjective weight
- BMI
- Quality of Life
- Emotional distress
- Sleep quality
- Physical activity
- Diet
- Alcohol consumption
- Smoking prevalence
- Pain attitudes
- Fear avoidance beliefs
- Health care utilisation

### Process measures and concomitant treatments

When indicated, data will be summarised per group. Again continuous variables will be summarised by use of standard measures of central tendency and dispersion, either mean and SD, or median and interquartile range. Categorical variables will be summarised by frequencies or denominators and percentages.

### Primary analysis

To examine between-group differences in the primary outcome (AUC – based on pain intensity score) we will use an independent sample Students t-test. The primary analysis will not adjust for known prognostic variables as covariates, but results adjusting for these will be presented as a sensitivity analysis (see below). Separate models will be estimated for each imputed dataset and the means and standard errors will be combined using Rubin’s method^25^. We will assess other model assumptions (homoscedasticity, normality) through inspecting appropriate residual plots, where serious violations are observed we will apply a rank-inverse normal transformation to the pain intensity score values. Dummy coded variables representing group allocation will be used to ensure blinding of the analyses. See Table 3 for details.

**Table 3:** Analyses of primary outcome.

**Analysis**	**Outcome**	**Intervention**	**Control**	**Difference**
Primary (ITT Multiple Imputation)	Area under the pain intensity curve (AUC)	mean(95%CI)	mean(95%CI)	mean(95%CI)
Sensitivity	Adjusted AUC*	mean(95%CI)	mean(95%CI)	mean(95%CI)
Secondary	Pain intensity score			
	Baseline	mean(SD)	mean(SD)	
	Week 2,	mean(SD)	mean(SD)	
	Week 6	mean(SD)	mean(SD)	
	Week 10	mean(SD)	mean(SD)	
	Week 14	mean(SD)	mean(SD)	
	Week 18	mean(SD)	mean(SD)	
	Week 22	mean(SD)	mean(SD)	
	Week 26	mean(SD)	mean(SD)	
	Weekly trend	mean(95%CI)	mean(95%CI)	mean(95%CI)

* Adjusted for baseline pain and duration, waiting time, previous surgery; BMI: physical activity and dietary intake; ITT: Intention to treat.

### Secondary analysis

Between group differences in the trajectory of pain intensities over the follow-up period will be examined using growth curve modelling. Hierarchical linear models will be used, with fixed effects for treatment group, time, and the interaction between the two. The model will include random subject-level intercepts and slopes. A linear growth trend will initially be assumed, and if not appropriate different functional forms for the trend will be applied (for example the square root transformation). If an appropriate functional form cannot be determined a flexible piecewise linear model will be used^26^. We will also investigate treatment effect heterogeneity that may exist in latent subgroups of participants through growth mixture models^27^. In these models a number of latent classes are specified that model the potential for participants to have different trajectory types, the functional forms identified from the previous growth curve analyses will inform the functional forms for this analysis. The model will include the following random effects that are all conditional trajectory class membership: intercept linear slope and quadratic slope. The random effects are influenced by the treatment group, so there will potentially be 3 lots of treatment effects (for each random effect) for each latent class.

Longitudinal generalised linear mixed models will be used to assess treatment effect on post randomisation secondary outcome measurements with random intercepts for individuals to account for correlation of repeated measures and an appropriate link function dependent on the type and distribution of the data. We will compare the adjusted mean change (continuous variables) or relative risks (dichotomous variables) in outcome from baseline to each time point between the treatment and control groups. A binomial distribution family (with log link) will be used for dichotomous outcomes (sleep quality, smoking prevalence), and a Poisson or negative-binomial distribution family (with a log link function) will be used for count outcomes (health care utilisation) based on assessment of data dispersion. T-tests will be used to test between group differences in variables collected only at 26 weeks (objective weight, BMI, waist circumference). See Table 4 for details.

**Table 4:** Secondary outcomes.

**Outcome**	**Intervention**	**Control**	**Intervention - control**
Disability and function (Trial 1: RMDQ / Trial 2: WOMAC)			
Baseline	mean (SD)	mean (SD)	mean (95% CI)
Week 6	mean (SD)	mean (SD)	mean (95% CI)
Week 26	mean (SD)	mean (SD)	mean (95% CI)
Overall	mean (SD)	mean (SD)	mean (95% CI)
Subjective weight			
Baseline	mean (SD)	mean (SD)	mean (95% CI)
Week 6	mean (SD)	mean (SD)	mean (95% CI)
Week 26	mean (SD)	mean (SD)	mean (95% CI)
Overall	mean (SD)	mean (SD)	mean (95% CI)
Objective weight			
Baseline^a^	mean (SD)	N/A	N/A
Week 26	mean (SD)	mean (SD)	mean (95% CI)
BMI			
Baseline	mean (SD)	mean (SD)	mean (95% CI)
Week 6	mean (SD)	mean (SD)	mean (95% CI)
Week 26	mean (SD)	mean (SD)	mean (95% CI)
Overall	mean (SD)	mean (SD)	mean (95% CI)
Waist circumference			
Week 26	mean (SD)	mean (SD)	mean (95% CI)
Quality of life (SF12v2, PCS)			
Baseline	mean (SD)	mean (SD)	mean (95% CI)
Week 6	mean (SD)	mean (SD)	mean (95% CI)
Week 26	mean (SD)	mean (SD)	mean (95% CI)
Overall	mean (SD)	mean (SD)	mean (95% CI)
Quality of life (SF12v2, MCS)			
Baseline	mean (SD)	mean (SD)	mean (95% CI)
Week 6	mean (SD)	mean (SD)	mean (95% CI)
Week 26	mean (SD)	mean (SD)	mean (95% CI)
Overall	mean (SD)	mean (SD)	mean (95% CI)
Perceived change in condition (GPE)			
Week 6	mean (SD)	mean (SD)	mean (95% CI)
Week 26	mean (SD)	mean (SD)	mean (95% CI)
Overall	mean (SD)	mean (SD)	mean (95% CI)
Emotional distress (DASS-21)			
Baseline	mean (SD)	mean (SD)	mean (95% CI)
Week 26	mean (SD)	mean (SD)	mean (95% CI)
Overall	mean (SD)	mean (SD)	mean (95% CI)
Poor sleep quality (item 6, Pittsburgh Sleep Quality Index)			
Baseline	n/N (%)	n/N (%)	OR (95% CI)
Week 6	n/N (%)	n/N (%)	OR (95% CI)
Week 26	n/N (%)	n/N (%)	OR (95% CI)
Overall	n/N (%)	n/N (%)	OR (95% CI)
Physical activity (mins MVPA/week)			
Baseline	mean (SD)	mean (SD)	mean (95% CI)
Week 6	mean (SD)	mean (SD)	mean (95% CI)
Week 26	mean (SD)	mean (SD)	mean (95% CI)
Overall	mean (SD)	mean (SD)	mean (95% CI)
Diet (Fruit, serves)			
Baseline	n/N (%)	n/N (%)	OR (95% CI)
Week 6	n/N (%)	n/N (%)	OR (95% CI)
Week 26	n/N (%)	n/N (%)	OR (95% CI)
Overall	n/N (%)	n/N (%)	OR (95% CI)
Diet (Vegetable, serves)			
Baseline	n/N (%)	n/N (%)	OR (95% CI)
Week 6	n/N (%)	n/N (%)	OR (95% CI)
Week 26	n/N (%)	n/N (%)	OR (95% CI)
Overall	n/N (%)	n/N (%)	OR (95% CI)
Diet (Discretionary foods, serves)			
Baseline	n/N (%)	n/N (%)	OR (95% CI)
Week 6	n/N (%)	n/N (%)	OR (95% CI)
Week 26	n/N (%)	n/N (%)	OR (95% CI)
Overall	n/N (%)	n/N (%)	OR (95% CI)
Alcohol consumption (AUDIT)			
Baseline	mean (SD)	mean (SD)	mean (95% CI)
Week 6	mean (SD)	mean (SD)	mean (95% CI)
Week 26	mean (SD)	mean (SD)	mean (95% CI)
Overall	mean (SD)	mean (SD)	mean (95% CI)
Smoking prevalence			
Baseline	n/N (%)	n/N (%)	OR (95% CI)
Week 6	n/N (%)	n/N (%)	OR (95% CI)
Week 26	n/N (%)	n/N (%)	OR (95% CI)
Overall	n/N (%)	n/N (%)	OR (95% CI)
Pain Attitudes (SOPA)			
Baseline	mean (SD)	mean (SD)	mean (95% CI)
Week 6	mean (SD)	mean (SD)	mean (95% CI)
Week 26	mean (SD)	mean (SD)	mean (95% CI)
Overall	mean (SD)	mean (SD)	mean (95% CI)
Fear avoidance beliefs (FABQ)			
Baseline	mean (SD)	mean (SD)	mean (95% CI)
Week 26	mean (SD)	mean (SD)	mean (95% CI)
Overall	mean (SD)	mean (SD)	mean (95% CI)
Health care utilisation (Medication use for back or knee pain)			
Baseline	n/N (%)	n/N (%)	OR (95% CI)
Week 6	n/N (%)	n/N (%)	OR (95% CI)
Week 26	n/N (%)	n/N (%)	OR (95% CI)
Overall	n/N (%)	n/N (%)	OR (95% CI)
Health care utilisation (Visits for back or knee pain)			
Baseline	n/N (%)	n/N (%)	OR (95% CI)
Week 6	n/N (%)	n/N (%)	OR (95% CI)
Week 26	n/N (%)	n/N (%)	OR (95% CI)
Overall	n/N (%)	n/N (%)	OR (95% CI)
Health care utilisation (Attended orthopaedic consultation for back or knee)			
Week 26	n/N (%)	n/N (%)	OR (95% CI)
Health care utilisation (Received surgery for back or knee)			
Week 26	n/N (%)	n/N (%)	OR (95% CI)

a Intervention group of low back pain patients (trial one) only.

RMDQ=Roland Morris Disability Questionnaire; WOMAC=Western Ontario and McMaster Universities Index; BMI=Body Mass Index; SF12.v2=Short Form Health Survey version 2; PCS=Physical Component Score; MCS=Mental Component Score; GPE=Global Perceived Effect; DASS-21=Depression Anxiety Stress Scale; MVPA=Moderate-to-Vigorous Physical Activity; AUDIT=Alcohol Use Disorders Identification Test; SOPA=Survey of Pain Attitudes; FABQ=Fear Avoidance Beliefs Questionnaire.

### Sensitivity analyses

Adjusting for prognostic variables:

The following variables hypothesised to effect outcome will be assessed by their inclusion as covariates in a linear regression model for the analysis of the primary outcome (AUC): baseline pain intensity, time since onset of pain, waiting time, BMI.

### Evaluation of adverse events

The Fisher exact test will be used to compare the incidence of any AEs between groups. This test will be used as the event rate of AEs is expected to be low.

## References

[1] Relton C, Torgerson D, O’Cathain A, Nicholl J (2010). Rethinking pragmatic randomised controlled trials: introducing
the “cohort multiple randomised controlled trial” design. BMJ.

[2] Williams A, Wiggers J, O’Brien KM, Wolfenden L, Yoong S, Campbell E (2016). A randomised controlled trial of a lifestyle behavioural
intervention for patients with low back pain, who are overweight or obese:
study protocol. BMC Musculoskelet Disord.

[3] O’Brien KM, Wiggers J, Williams A, Campbell E, Wolfenden L, Yoong S (2016). Randomised controlled trial of referral to a telephone-based
weight management and healthy lifestyle programme for patients with knee
osteoarthritis who are overweight or obese: a study protocol. BMJ Open.

[4] O’Hara B, Phongsavan P, Venugopal K, Eakin E, Eggins D, Caterson H (2012). Effectiveness of Australia’s get healthy information and coaching
service: translational research with population wide impact. Prev Med.

[5] Krismer M, van Tulder M (2007). Strategies for prevention and management of musculoskeletal
conditions. Low back pain (non-specific). Best Pract Res Clin Rheumatol.

[6] Jensen MP, Turner JA, Romano JM, Fisher LD (1999). Comparative reliability and validity of chronic pain intensity
measures. Pain.

[7] Ware JE, Kosinski M, Turner-Bowker DM, Gandek B (2002). User’s manual for the SF-12v2 health survey (with a supplement
documenting SF-12 health survey).

[8] My Hospitals About the data: elective surgery waiting times.

[9] Roland M, Morris R (1983). A study of the natural history of back pain. Part 1: development
of a reliable and sensitive measure of disability in low back
pain. Spine (Phila Pa 1976).

[10] Bellamy N (2009). WOMAC user guide IX.

[11] International Society for the Advancement of Kinanthropometry –
ISAK (2001). International standards for anthropometric assessment.

[12] National Institutes of Health, National Heart, Lung and Blood
Institute – NHLBI (2000). The practical guide: identification, evaluation, and treatment of
overweight and obesity in adults.

[13] Kamper SJ, Ostelo RWJG, Knol DL, Maher CG, de Vet HCW, Hancock MJ (2010). Global Perceived Effect scales provided reliable assessments of
health transition in people with musculoskeletal disorders, but ratings are
strongly influenced by current status. J Clin Epidemiol.

[14] Lovibond SH, Lovibond PF (1995). Manual for the depression anxiety stress scales.

[15] Buysse DJ, Reynolds CF, Monk TH, Berman SR, Kupfer DJ (1989). The Pittsburgh sleep quality index: a new instrument for
psychiatric practice and research. Psychiatry Res.

[16] Australian Institute of Health and Welfare – AIHW (2003). The active Australia survey: a guide and manual for implementation,
analysis and reporting.

[17] Centre for Epidemiology and Research (2014). NSW Population Health Survey.

[18] Babor T, Higgins-Biddle J, Saunders J, Monteiro M (1992). AUDIT: the alcohol use disorders identification test: guidelines for use
in primary care.

[19] Shiri R, Karppinen J, Leino-Arjas P, Solovieva S, Viikari-Juntura E (2010). The association between smoking and low back pain: a
meta-analysis. Am J Med.

[20] Jensen MP, Karoly P, Huger R (1987). The development and preliminary validation of an instrument to
assess patients’ attitudes toward pain. J Psychosom Res.

[21] Waddell G, Newton M, Henderson I, Somerville D, Main CJ (1993). A Fear-Avoidance Beliefs Questionnaire (FABQ) and the role of
fear-avoidance beliefs in chronic low back pain and
disability. Pain.

[22] Proschan MA, Waclawiw MA (2000). Practical guidelines for multiplicity adjustment in clinical
trials. Control Clin Trials.

[23] Christensen R, Bartels EM, Astrup A, Bliddal H (2007). Effect of weight reduction in obese patients diagnosed with knee
osteoarthritis: a systematic review and meta‐analysis. Ann Rheum Dis.

[24] Molenberghs G, Kenward M (2007). Missing data in clinical studies.

[25] Marshall A, Altman DG, Holder RL, Royston P (2009). Combining estimates of interest in prognostic modelling studies
after multiple imputation: current practice and guidelines. BMC Med Res Methodol.

[26] Gallop R, Dimidjian S, Atkins D, Muggeo V (2011). Quantifying treatment effects when flexibly modeling individual
change in a nonlinear mixed effects model. J Data Sci.

[27] Muthen B, Brown C, Hunter A, Cook I, Leuchter A, Shrout P, Keyes K, Ornstein K (2011). General approaches to analysis of course: applying growth mixture
modeling to randomized trials of depression medication. Causality and psychopathology: finding the determinants of disorders and
their cures.

